# Supplementary material for: IL-1β promotes adipogenesis by directly targeting adipocyte precursors
Source: Nat Commun. 2024 Sep 11;15:7957. doi: 10.1038/s41467-024-51938-x (PMC11390900; doi:10.1038/s41467-024-51938-x)
Supplement: Supplementary file 1 — Supplementary Information [file 41467_2024_51938_MOESM1_ESM.pdf]

IL-1β promotes adipogenesis by directly targeting adipocyte precursors

Supplementary Information

This file includes Supplementary Figures 1-11 and Supplementary Table 1

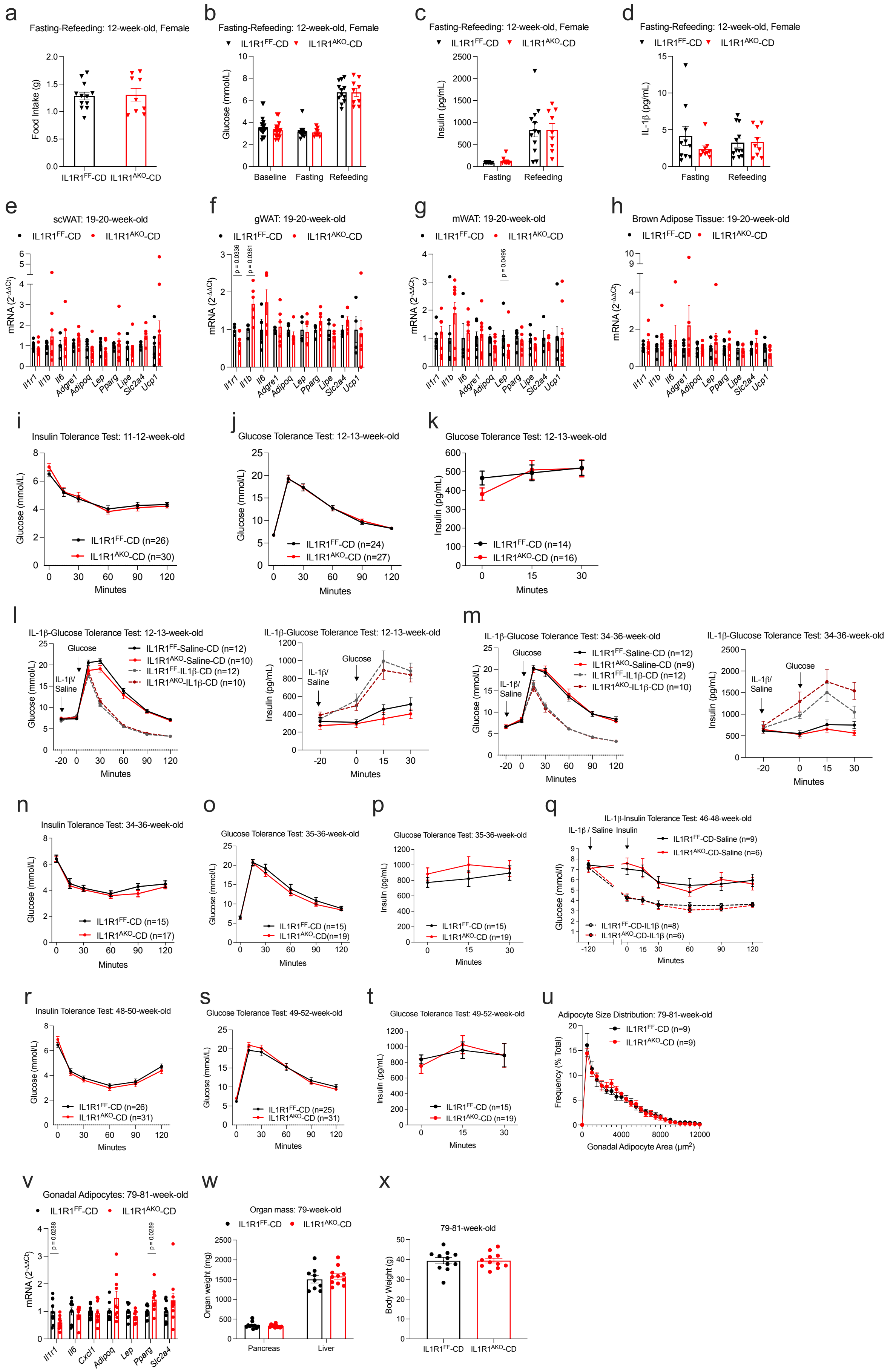

Supplementary Fig. 1. Chow-fed IL1R1^{AKO} mice have no metabolic phenotype (related to Fig. 1). (**a-d**) Food intake (**a**), glucose (**b**), insulin (**c**), and IL-1β (**d**) levels during fasting-refeeding experiment in 12-week-old chow-fed female mice. Food intake, refeeding glucose, insulin, and IL-1β: n=12 IL1R1^{FF}, n=9 IL1R1^{AKO}. Fasting glucose, insulin, and IL-1β: n=10 IL1R1^{FF}, n=9 IL1R1^{AKO}. Glucose baseline: n=22 IL1R1^{FF}, n=18 IL1R1^{AKO}.

(**e**-**h**) Relative mRNA expression in scWAT (**e**), gWAT (**f**), mWAT (**g**), and brown adipose tissue (BAT) (**h**) of 19-20-week-old chow-fed male mice. n=4 IL1R1^{FF}, n=6 IL1R1^{AKO} for *Il6* and *Slc2a4*; n=6 IL1R1^{FF}, n=9 IL1R1^{AKO} for remaining genes (**e**, **g**). n=4 IL1R1^{FF}, n=9 IL1R1^{AKO} for *Il1r1*; n=4 IL1R1^{FF}, n=6 IL1R1^{AKO} for remaining genes (**f**). n=4 IL1R1^{FF}, n=5 IL1R1^{AKO} for *Il6* and *Slc2a4*; n=6 IL1R1^{FF}, n=8 IL1R1^{AKO} for remaining genes (**h**).
(**i**-**k**) Insulin tolerance test (**i**), glucose tolerance test (**j**), and insulin levels (**k**) in 11-12- (**i**) and 12-13-week-old (**j**-**k**) male mice. n=26 IL1R1^{FF}, n=30 IL1R1^{AKO} (**i**), n=24 IL1R1^{FF}, n=27 IL1R1^{AKO} (**j**), n=14 IL1R1^{FF} and n=16 IL1R1^{AKO} (**k**).
(**l**, **m**) Concentration of circulating glucose and insulin during a glucose tolerance test in 12-13- (**l**) and 34-36-week-old (**m**) male chow-fed mice. An injection of saline or IL-1β (1 µg/kg bw) was administrated 20 minutes before the glucose bolus. n=12 IL1R1^{FF}, n=10 IL1R1^{AKO} (**l**). n=12 IL1R1^{FF}, n=9 IL1R1^{AKO}-saline, n=10 IL1R1^{AKO}-IL-1β (**m**).
(**n**-**p**) Insulin tolerance test (**n**), glucose tolerance test (**o**), and insulin levels (**p**) in 34-36- (**n**) and 35-36-week-old (**o**, **p**) male mice. n=15 IL1R1^{FF}, n=17 IL1R1^{AKO} (**n**). n=15 IL1R1^{FF}, n=19 IL1R1^{AKO} (**o**, **p**).
(**q**) Concentration of circulating glucose during an insulin tolerance test in 46-48-week-old male chow-fed mice. An injection of saline or IL-1β (1 µg/kg bw) was administrated 20 minutes before the insulin bolus (1.4 U/kg bw). n=9 IL1R1^{FF}-saline, n=8 IL1R1^{FF}- IL-1β, n=6 IL1R1^{AKO}.
(**r**-**t**) Insulin tolerance test (**r**), glucose tolerance test (**s**), and insulin levels (**t**) in 48-50- (**r**) and 49-52-week-old (**s**, **t**) chow-fed male mice. n=26 IL1R1^{FF}, n=31 IL1R1^{AKO} (**r**). n=25 IL1R1^{FF}, n=31 IL1R1^{AKO} (**s**). n=15 IL1R1^{FF}, n=19 IL1R1^{AKO} (**t**).
(**u**-**x**) Cell size distribution (**u**), relative mRNA expression in adipocytes from gWAT (**v**), and organ and body weight (**w**-**x**) in 79-81-week-old chow-fed male mice. n=9 (**u**). n=10 IL1R1^{FF}, n=9 IL1R1^{AKO} for *Il6* and *Lep*, n=10 for remaining genes (**v**). n=10 IL1R1^{FF}, n=11 IL1R1^{AKO} for pancreas. n=9 IL1R1^{FF}, n=11 IL1R1^{AKO} for liver (**w**). n=11 (**x**).
n = biological replicates. Data are shown as individual measurements and mean ± SEM. Statistical analyses were performed by two-way ANOVA and Šidák's multiple comparison test (**i**-**u**) or unpaired nonparametric two-tailed Mann-Whitney U test (all other panels). Source data are provided as a Source Data File.

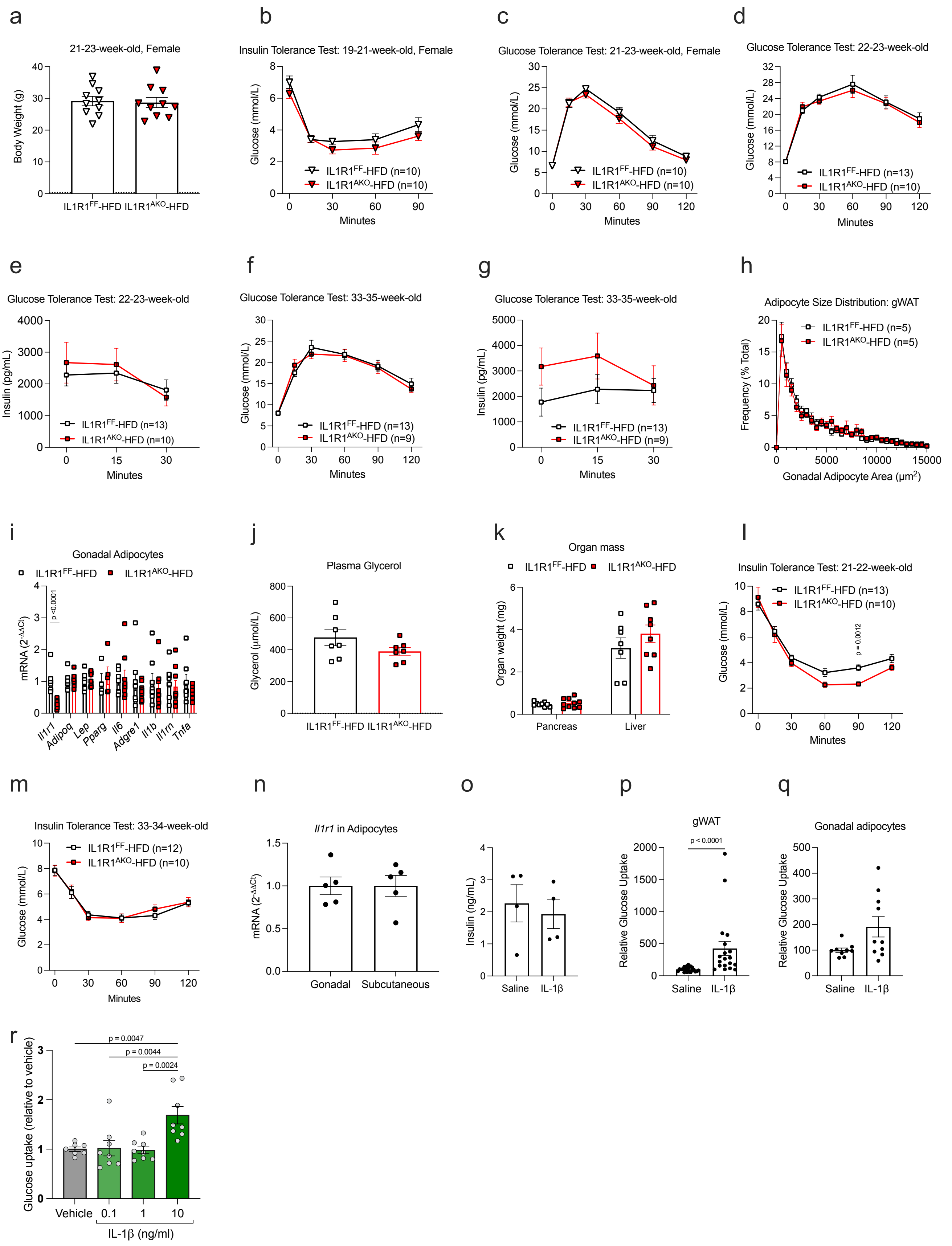

Supplementary Fig. 2. Female HFD-fed IL1R1^{AKO} mice have no metabolic phenotype while HFD-fed IL1R1^{AKO} male mice show a mild insulin phenotype (related to Fig. 1).

(**a**-**c**) Body weight (**a**), insulin tolerance test (**b**), and glucose tolerance test (**c**) in 21-23- (**a**, **c**) and 19-21-week-old (**b**) HFD-fed female mice (n=10).

(**d**, **e**) Glucose tolerance test (**d**) and insulin levels (**e**) in 22-23-week-old HFD-fed male mice. n=13 IL1R1^{FF}, n=10 IL1R1^{AKO}.

(**f**, **g**) Glucose tolerance test (**f**) and insulin levels (**g**) in 33-35-week-old HFD-fed male mice. n=13 IL1R1^{FF}, n=9 IL1R1^{AKO}.

(**h**-**k**) Cell size distribution (**h**), relative mRNA expression in adipocytes from gWAT (**i**), plasma glycerol (**j**) and organ weight (**k**) of 40-45-week-old HFD-fed male mice. n=5 (**h**). n=4 IL1R1^{FF}, n=5 IL1R1^{AKO} for *Pparg*; n=8 IL1R1^{FF}, n=9 IL1R1^{AKO} for remaining genes (**i**). n=7 (**j**). n=9 IL1R1^{FF}, n=10 IL1R1^{AKO} for pancreas. n=7 IL1R1^{FF}, n=8 IL1R1^{AKO} for liver (**k**).

(**l**, **m**) Insulin tolerance test in 21-22- (**l**) and 33-34-week-old (**m**) HFD-fed male mice. n=13 IL1R1^{FF}, n=10 IL1R1^{AKO} (**l**). n=12 IL1R1^{FF}, n=10 IL1R1^{AKO} (**m**).
(**n**) Relative mRNA levels of *Il1r1* in adipocytes from gWAT and scWAT of 12-week-old male mice (n = 5).
(**o-q**) Insulin levels (**o**) and glucose uptake in gWAT (**p**) and gonadal adipocytes (**q**) of WT mice treated with saline or IL-1β (1 μg/kg bw) 18 minutes prior to euthanasia and tissue harvesting. n=4 (**o**). n=21 saline, n=19 IL-1β (**p**). n=9 saline, n=10 IL-1β (**q**).
(**r**) Glucose uptake in *in vitro* differentiated human adipocytes treated with indicated concentrations of IL-1β for 2 h (n= 4 independent experiments with 1-2 replicates (7 datapoints for vehicle and 8 for the rest)).
HFD-feeding started at 10 weeks of age. n=biological replicates (**a**-**q**). Data are shown as individual measurements and mean ± SEM. Statistical analyses were performed by unpaired nonparametric two-tailed Mann-Whitney U test (**a**, **i**-**k**, **n**-**q**), one-way ANOVA and Tukey's multiple comparisons test (**r**), or two-way ANOVA and Šidák's multiple comparisons test (all other panels). Source data are provided as a Source Data File.

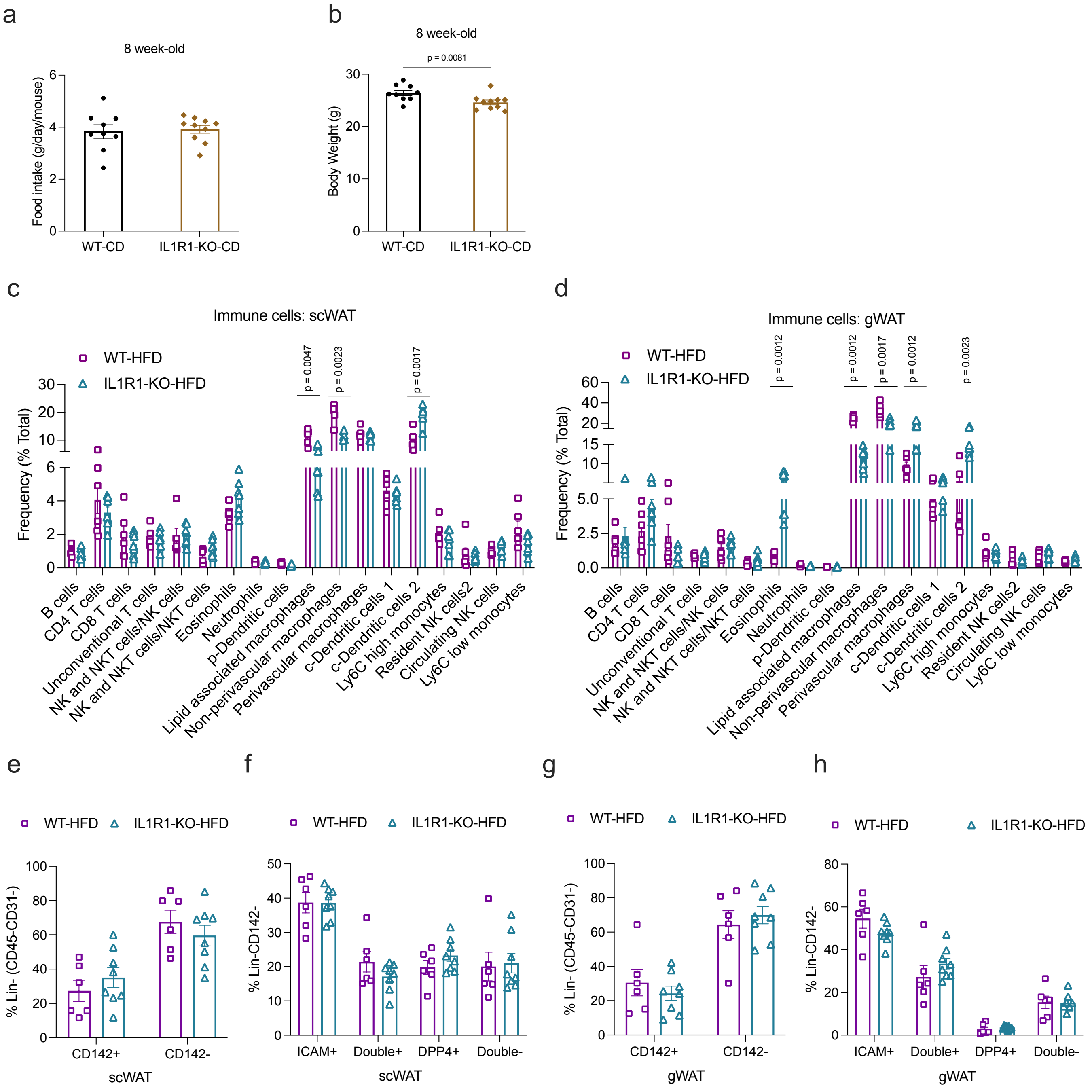

Supplementary Fig. 3. IL1R1 ablation does not affect food intake nor adipocyte progenitor subpopulations but alters myeloid cell proportions in WAT (Related to Fig. 2).

(**a**, **b**) Food intake (**a**) and body weight (**b**) of 8-week-old male chow-fed IL1R1-KO and WT mice. n=9 IL1R1-WT, n=10 IL1R1-KO.

(**c**, **d**) Proportions of immune cell subpopulations in scWAT (**c**) and gWAT (**d**) of 17-week-old HFD-fed IL1R1-KO and WT mice. n=6 IL1R1-WT, n=7 IL1R1-KO.

(**e**-**h**) Proportions of adipocyte progenitor subpopulations in scWAT (**e**, **f**) and gWAT (**g**, **h**) of 9-week-old IL1R1-KO and WT mice HFD-fed for 1 week. n=6 IL1R1-WT, n=8 IL1R1-KO.

Data are shown as individual measurements and mean ± SEM. Statistical analyses were performed by unpaired nonparametric two-tailed Mann-Whitney U test. Source data are provided as a Source Data File.

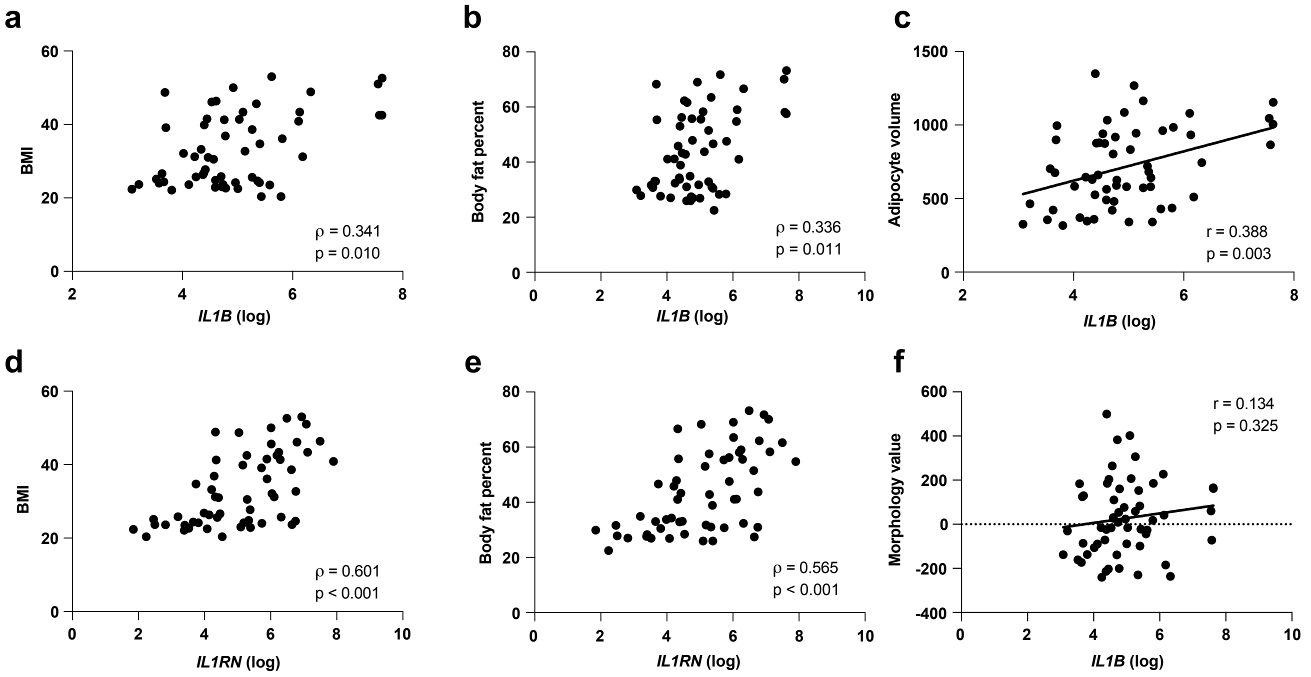

Supplementary Fig. 4. Characterization of the IL-1β system in human scWAT (related to Figure 3).
(**a**-**c**) Pearson (r coefficient) or Spearman (ρ coefficient) correlations between human scWAT mRNA expression of *IL1B* and BMI (**a**), body fat percent (**b**), or adipocyte volume (**c**) (n=56).
(**d**, **e**) Spearman (ρ coefficient) correlations between human scWAT mRNA expression of *IL1RN* and BMI (**d**) or body fat percent (**e**) (n=56).
(**f**) Pearson (r coefficient) correlation between human scWAT mRNA expression of *IL1B* and WAT morphology value (n=56).
Two-tailed p-values.

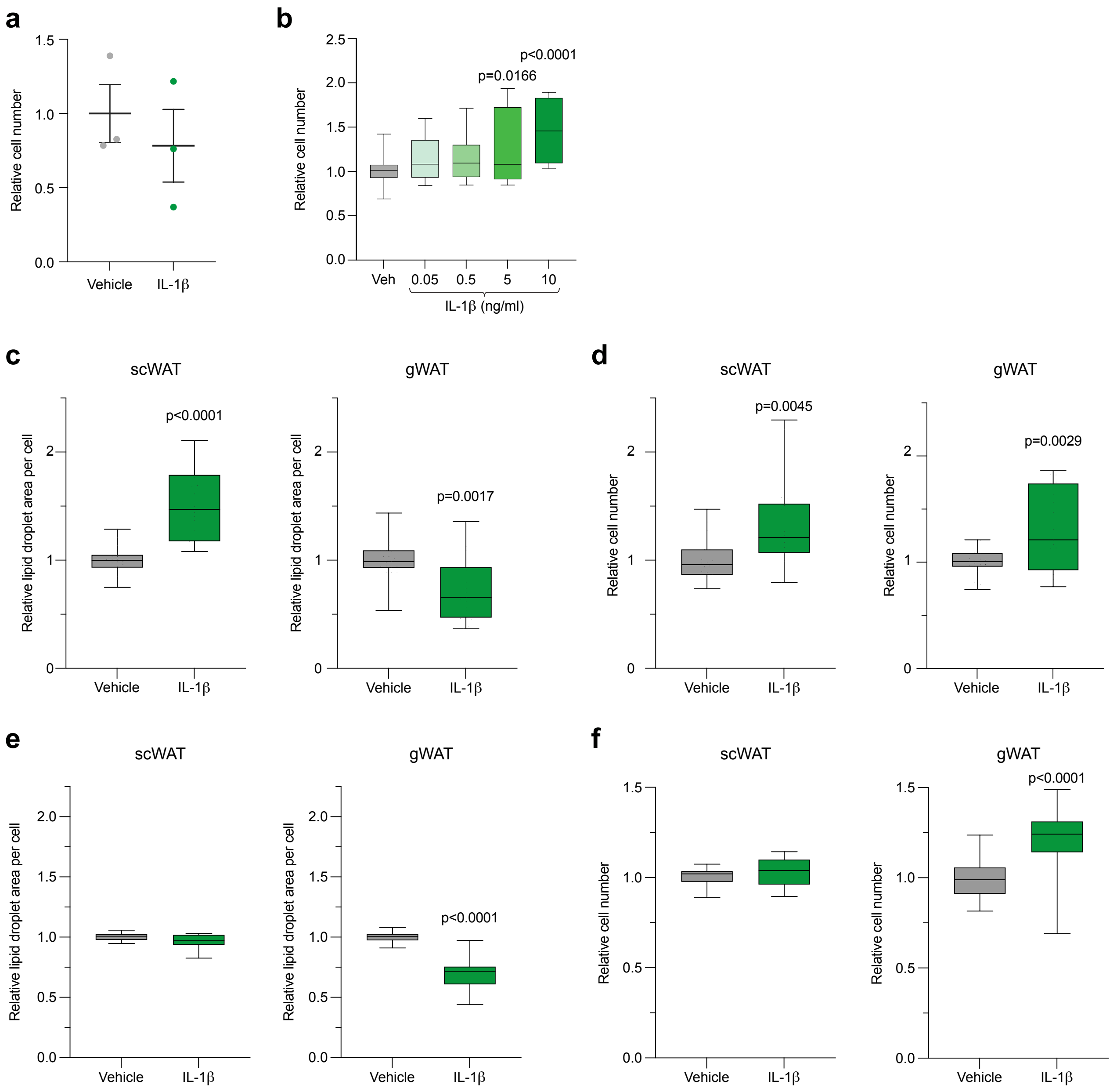

Supplementary Fig. 5. Cell number and lipid droplet accumulation in IL-1β-treated WAT-derived progenitors (related to Fig. 4).
(**a**, **b**) Cell number in experiments described in Fig. 4b (**a**, n = 3 donors) and 4d (**b**, n = 3 independent experiments with ≥ 4 replicates (35 datapoints in vehicle and 12 in rest)).
(**c**, **d**) Lipid droplet accumulation (**c**) and cell number (**d**) in CD45^{-} stromal vascular cells from scWAT and gWAT of 8–9-week-old male mice, differentiated with or without IL-1β (10 ng/ml) (n = 5 independent experiments, 3 pooled mice with 2-4 replicates per experiment (18 (scWAT) and 19 (gWAT) datapoints)).
(**e**, **f**) Lipid droplet accumulation (**e**) and cell number (**f**) in CD45^{-} stromal vascular cells from scWAT and gWAT of male *ob*/*ob* mice, differentiated with or without IL-1β (10 ng/ml) (n = 3 independent experiments with 1-16 replicates (scWAT: 11 (vehicle) and 9 (IL-1β) datapoints; gWAT: 26 (vehicle) and 23 (IL-1β) datapoints)).
Statistical analyses by paired (**a**) or unpaired (**c-f**) two-tailed *t* test, or one-way ANOVA and Dunnett's multiple comparisons test compared to vehicle (**b**). Data are represented as individual measurements and mean ± SEM or box-and-whisker plots (line inside box = median; box limits = first and third quartiles; whisker ends = minima and maxima). Source data are provided as a Source Data File.

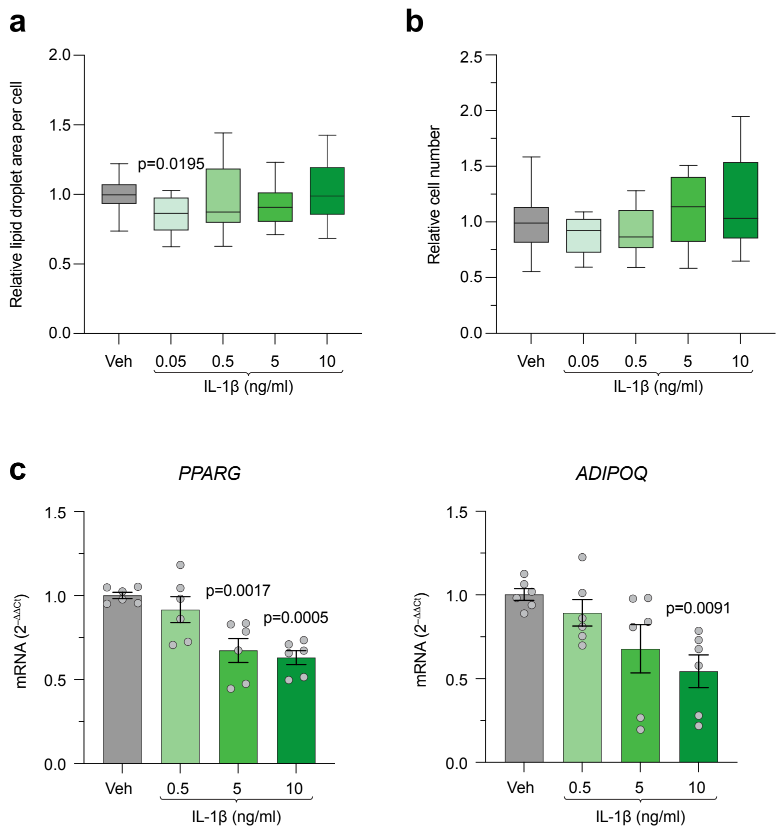

Supplementary Fig. 6. Effect of long-term IL-1β treatment on adipogenesis (related to Fig. 5).
(**a**-**b**) Lipid droplet accumulation (**a**) and cell number (**b**) in hASCs differentiated for 13 days with IL-1β (10 ng/ml) (n = 3 independent experiments with ≥ 4 replicates (35 datapoints in vehicle and 12 in the rest)).
(c) *PPARG* and *ADIPOQ* expression in hASCs differentiated for 13 days with IL-1β (10 ng/ml) (n = 3 independent experiments with 2 replicates).
Statistical analyses by one-way ANOVA and Dunnett's multiple comparisons test compared to vehicle. Data are represented as box-and-whisker plots (line inside box = median; box limits = first and third quartiles; whisker ends = minima and maxima) or individual data points and mean ± SEM. Source data are provided as a Source Data File.

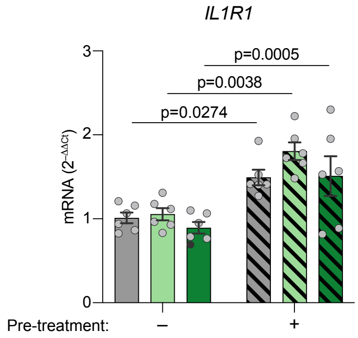

Supplementary Fig. 7. *IL1R1* expression in response to pre-treatment (related to Fig. 6).
IL1R1 expression in the same experiment as described in Fig. 6c-d. Conditions are color/pattern coded as in Fig. 6b-d. Statistical analyses by two-way ANOVA and Šidák's and Tukey's multiple comparisons tests (n = 3 independent experiments with 2 replicates). Data are represented as individual data points and mean ± SEM. Source data are provided as a Source Data File.

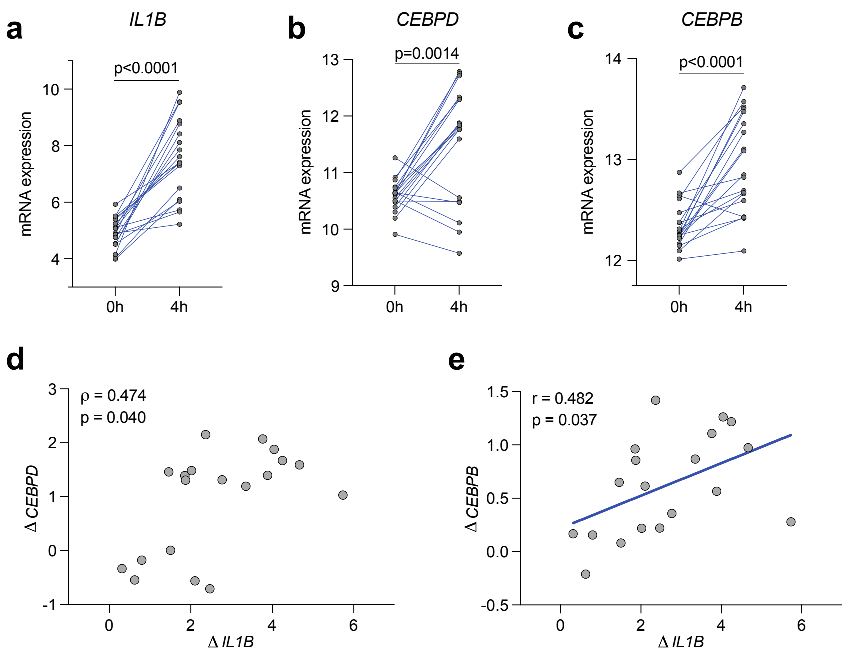

Supplementary Fig. 8. *IL1B*, *CEBPD* and *CEBPB* are upregulated in human scWAT postprandially.
(**a**-**c**) Normalized expression values of *IL1B* (**a**), *CEBPD* (**b**), and *CEBPB* (**c**) at baseline (0 h) and 4 h after a meal in scWAT from middle-aged males, analysed from a publicly available microarray dataset (n = 19). Paired two-tailed t test (**a**, **c**) or Wilcoxon matched-pairs signed rank test (**b**).
(**d**, **e**) Spearman (ρ coefficient) (**d**) or Pearson (r coefficient) (**e**) correlations between the absolute postprandial change (4 h – 0 h) in expression values of *IL1B* and *CEBPD* (**d**) or *CEBPB* (**e**) in the same dataset as described in (**a**-**c**).

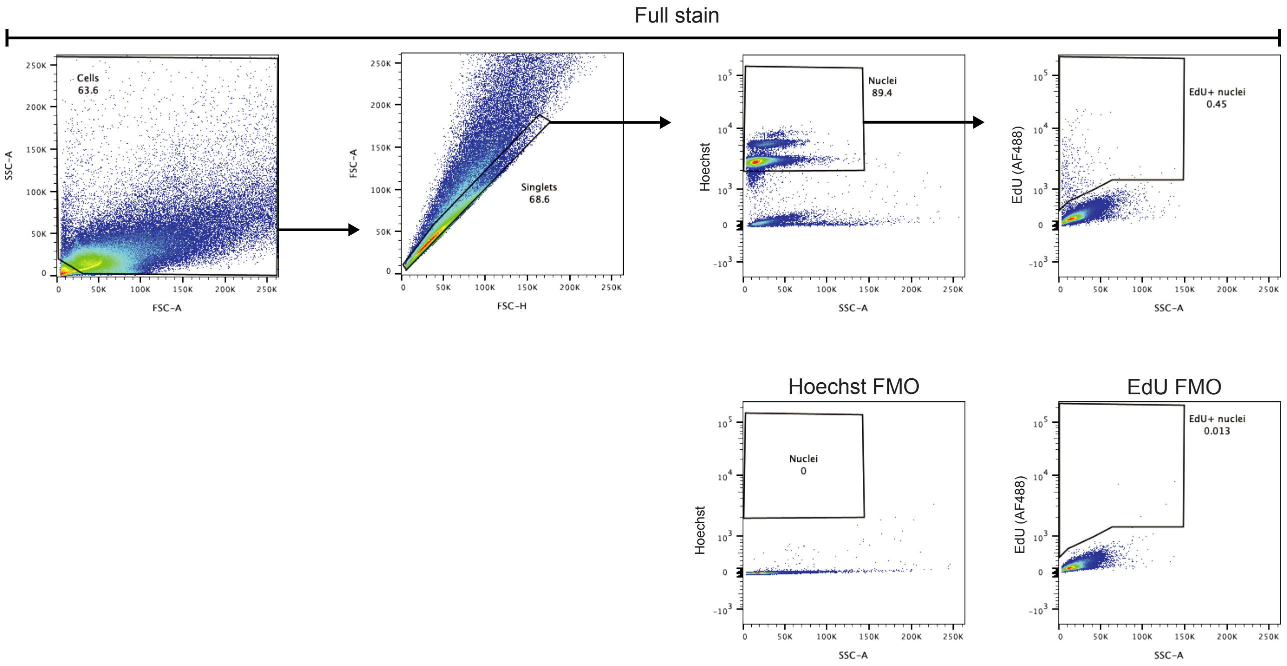

Supplementary Fig. 9. FACS gating strategy for analysis of EdU⁺ adipocyte nuclei.
Representative FACS gating scheme to identify EdU⁺ adipocyte nuclei in WAT of mice shown in Fig. 2r, s, u, v.

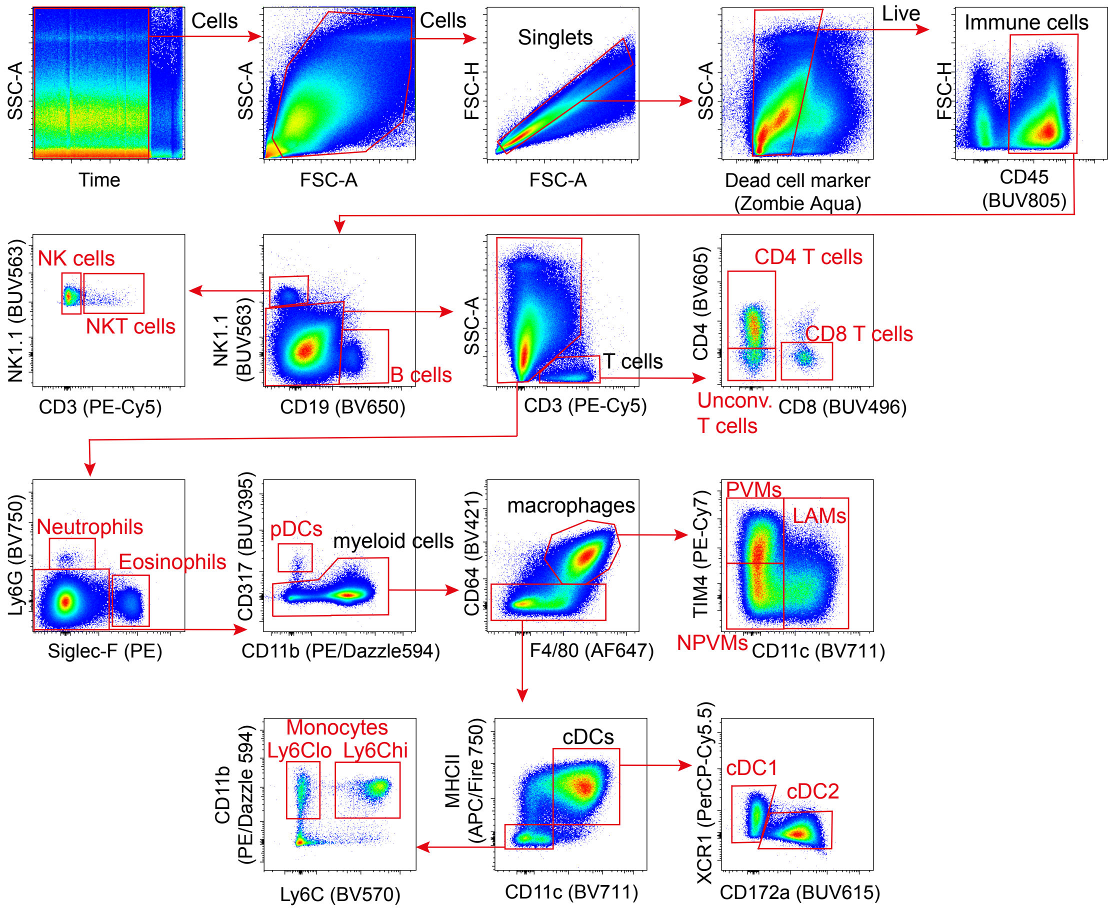

Supplementary Fig. 10. FACS gating strategy for analysis of immune cell populations.
Representative FACS gating scheme to identify immune cell populations in the stromal vascular fraction of WT and IL1R1-KO mice shown in Supplementary Fig. 3c, d.

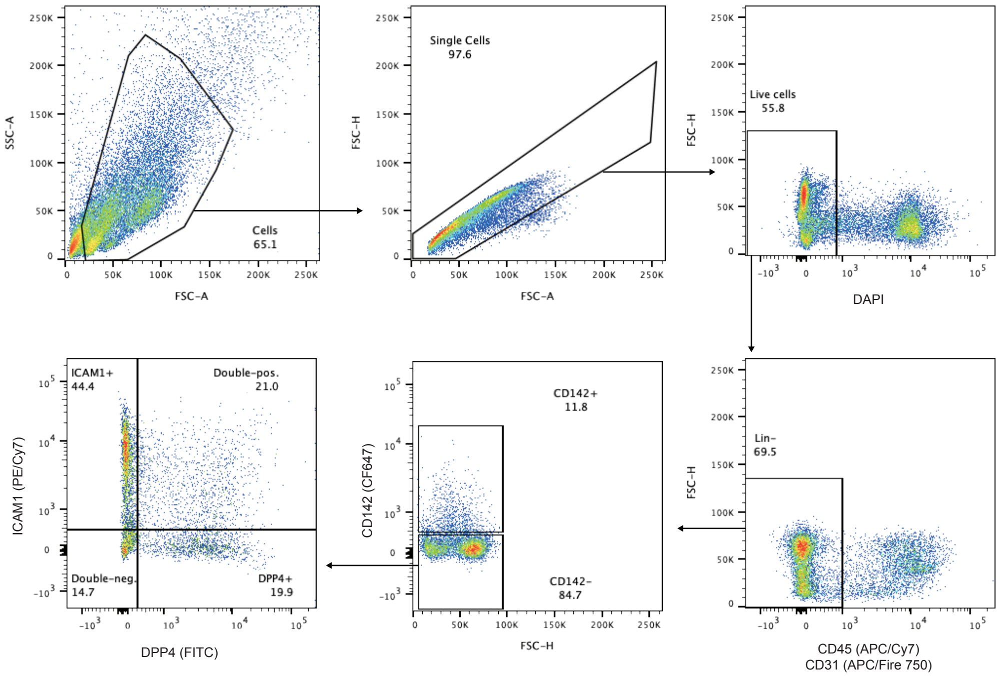

Supplementary Fig. 11. FACS gating strategy for analysis of progenitor subpopulations.
Representative FACS gating scheme to identify progenitor subpopulations in the stromal vascular fraction of WT and IL1R1-KO mice shown in Supplementary Fig. 3e-h.

Supplementary Table 1. List of reagents

Antibodies for FACS	SOURCE	IDENTIFIER
Anti-mouse antibodies for immune cell panel		
Anti-CD206-AF488 (Clone C068C2) (1:100)	Biolegend	Cat#141710
Anti-XCR1-PerCP-Cy5.5 (Clone ZET) (1:100)	Biolegend	Cat#148208
Anti-CD317-BUV395 (Clone 927) (1:100)	BD Biosciences	Cat#747602
Anti-CD8-BUV496 (Clone 53-6.7) (1:100)	BD Biosciences	Cat#569181
Anti-NK1.1-BUV563 (Clone PK136) (1:50)	BD Biosciences	Cat#741233
Anti-CD172a-BUV615 (Clone P84) (1:100)	BD Biosciences	Cat#751214
Anti-CD49b-BUV661 (Clone HMa2) (1:200)	BD Biosciences	Cat#741523
Anti-CD45-BUV805 (Clone 30-F11) (1:100)	BD Biosciences	Cat#568336
Anti-F4/80-AF647 (Clone Cl:A3-1) (1:10)	Bio-Rad Laboratories	Cat#MCA497A64 7
Anti-MHCII-APC/Fire750 (Clone M5/114.15.2) (1:400)	Biolegend	Cat#107651
Anti-CD64-BV421 (Clone X54-5/7.1) (1:50)	Biolegend	Cat#139309
Anti-Ly6C-BV570 (Clone HK1.4) (1:100)	Biolegend	Cat#128030
Anti-CD4-BV605 (Clone RM4-5) (1:50)	Biolegend	Cat#100547
Anti-CD19-BV650 (Clone 6D5) (1:100)	Biolegend	Cat#115541
Anti-CD11c-BV711 (Clone HL3) (1:25)	BD Biosciences	Cat#563048
Anti-Ly6G-BV750 (Clone 1A8) (1:200)	BD Biosciences	Cat#747072
Anti-CD49a-BV786 (Clone Ha31/8) (1:100)	BD Biosciences	Cat#740919
Anti-Siglec-F-PE (Clone E50-2440) (1:100)	BD Biosciences	Cat#562068
Anti-CD11b-PE/Dazzle594 (Clone M1/70) (1:200)	Biolegend	Cat#101256
Anti-CD3e-PE-Cy5 (Clone 145-2C11) (1:50)	BD Biosciences	Cat#553065
Anti-TIM4-PE-Cy7 (Clone RMT4-54) (1:50)	Biolegend	Cat#130009
Anti-CD16/CD32 (Clone 2.4G2) (Mouse BD Fc Block™)	BD Biosciences	Cat#553142
Anti-mouse antibodies for progenitor subtype panel		

Anti-CD45-APC/Cy7 (Clone 30-F11) (1:1000)	BioLegend	Cat#103116
Anti-CD31-APC/Fire 750 (Clone MEC13.3) (1:1000)	BioLegend	Cat#102528
Anti-CD142 (Polyclonal) (1:20)	Novus Biologicals	Cat#NBP2-15139
Anti-CD54 (ICAM-1)-PE/Cy7 (Clone YN1/1.7.4) (1:100)	BioLegend	Cat#116122
Anti-CD26 (DPP-4)-FITC (Clone H194-112) (1:200)	BioLegend	Cat#137806
FcR Blocking reagent, mouse (1:10)	Miltenyi Biotec	Cat#130-092-575
Anti-human antibodies for progenitor sorting		
Anti-CD31-PE-Cy7 (Clone WM59) (1:12.5)	BD Biosciences	Cat#563651
Anti-CD34-PE-CF594 (Clone 563) (1:250)	BD Biosciences	Cat#562449
Anti-CD45-AF700 (Clone HI30) (1:100)	BD Biosciences	Cat#560566

**Antibodies for other applications**		
CD45 MicroBeads, mouse (Clone 30F11.1)	Miltenyi Biotec	Cat#130-052-301
Anti-C/EBPδ (Clone C-6) (Western Blot: 1:500; ChIP: 3 μg)	Santa Cruz Biotechnology	Cat#sc-365546
Anti-C/EBPβ (LAP) (Polyclonal) (Western Blot 1:1000)	Cell Signaling	Cat#3087
Anti-Lamin A/C (Clone 4C11) (Western Blot 1:1000)	Cell Signaling	Cat#4777
Anti-P-CREB (S133) (Clone 87G3) (Western Blot 1:1000)	Cell Signaling	Cat#9198
Anti-CREB (Clone 48H2) (Western Blot 1:1000)	Cell Signaling	Cat#9197
Anti-rabbit IgG, HRP-linked (Western Blot 1:10000)	Cell Signaling	Cat#7074
Anti-mouse IgG, HRP-linked (Polyclonal) (Western Blot 1:10000)	Invitrogen	Cat#G21040

Anti-C/EBPβ (Polyclonal) (ChIP: 3 μg)	Santa Cruz Biotechnology	Cat#sc-150
Normal Mouse IgG (Polyclonal) (ChIP: 3 μg)	EMD Millipore	Cat#12-371
Normal Rabbit IgG (Polyclonal) (ChIP: 3 μg)	EMD Millipore	Cat#12-370

**Oligonucleotides**		
PPARG (Taqman probe)	Applied Biosystems	Hs01115513_m1
ADIPOQ (Taqman probe)	Applied Biosystems	Hs00605917_m1
PLIN1 (Taqman) probe	Applied Biosystems	Hs00193510_m1
PPIA (Taqman probe)	Applied Biosystems	Hs041994521_s1
FABP4 (Taqman probe)	Applied Biosystems	Hs01086177_m1
FASN (Taqman probe)	Applied Biosystems	Hs01005622_m1
ACACB (Taqman probe)	Applied Biosystems	Hs00153715_m1
CCL2 (Taqman probe)	Applied Biosystems	Hs00234140_m1
IL6 (Taqman probe)	Applied Biosystems	Hs00985639_m1
CSF3 (Taqman probe)	Applied Biosystems	Hs00738432_g1
CCL5 (Taqman probe)	Applied Biosystems	Hs00174575_m1
CXCL12 (Taqman probe)	Applied Biosystems	Hs03676656_mH
NFKB1 (Taqman probe)	Applied Biosystems	Hs00765730_m1
IL1R1 (Taqman probe)	Applied Biosystems	Hs00168392_m1
PPARG (SYBR Green primer)	Sigma-Aldrich	F:CCCAGAAAGCGATTCCTTCAC; R:AGCTGATCCCAAAGTTGGTGG
ADIPOQ (SYBR Green primer)	Sigma-Aldrich	F:GGTCTTATTGGTCCTAAGGG; R:GTAGAAGATCTTGGTAAAGCG

PLIN1 (SYBR Green primer)	Sigma-Aldrich	F:TGGAGACTGAGGAGAACAAG; R:ATGTCACAGCCGAGATGG
PPIA (SYBR Green primer)	Sigma-Aldrich	F:CCCACCGTGTTCTTCGACATT; R:GGACCCGTATGCTTTAGGATGA
FABP4 (SYBR Green primer)	Sigma-Aldrich	F:CAAGAGCACCATAACCTTAG; R:CTCGTTTTCTCTTTATGGTGG
CCL2 (SYBR Green primer)	Sigma-Aldrich	F:AGGTGACTGGGGCATTGAT; R:GCCTCCAGCATGAAAGTCTC
IL6 (SYBR Green primer)	Sigma-Aldrich	F:ACTCACCTCTTCAGAACGAATTG; R:CCATCTTTGGAAGGTTCAGGTTG
CEBPD (SYBR Green primer)	Sigma-Aldrich	F:CAGACTTTTCAGACAAACCC; R:TTTCGATTTCAAATGCTGC
CEBPB (SYBR Green primer)	Sigma-Aldrich	F:ATAAACTCTCTGCTTCTCCC; R:CCGTAGGAACATCTTTAAGC

CEBPA (SYBR Green primer)	Sigma-Aldrich	F:AGCCTTGTTTGTACTGTATG; R:AAAATGGTGGTTTAGCAGAG
DDIT3 (SYBR Green primer)	Sigma-Aldrich	F:CTTTTCCAGACTGATCCAAC; R:GATTCTTCCTCTTCATTTCCAG
Il1r1 (SYBR Green primer)	Microsynth	F:GCACGCCCAGGAGAATATGA; R:AGAGGACACTTGCGAATATCAA
Adipoq (SYBR Green primer)	Microsynth	F:TGACGACACCAAAAGGGCTC; R:CACAAGTTCCCTTGGGTGGA
Fasn (SYBR Green primer)	Microsynth	F:GCTGCGGAAACTTCAGGAAAT; R:AGAGACGTGTCACTCCTGGACTT
Lep (SYBR Green primer)	Microsynth	F:GAGACCCCTGTGTCGGTTC; R:GAGACCCCTGTGTCGGTTC
Plin1 (SYBR Green primer)	Microsynth	F:CTGTGTGCAATGCCTATGAGA;

		R:CTGGAGGGTATTGAAGAGCCG
Hprt (SYBR Green primer)	Microsynth	F:TCAGTCAACGGGGGACATAAA; R:GGGGCTGTACTGCTTAACCAG
Gapdh (SYBR Green primer)	Microsynth	F:AGGTCGGTGTGAACGGATTTG; R:TGTAGACCATGTAGTTGAGGTCA
Pparg (SYBR Green primer)	Microsynth	F:GCCTATGAGCACTTCACAAGAAAT; R:GGAATGCGAGTGGTCTTCCA
Il6 (SYBR Green primer)	Microsynth	F:GCCTTCTTGGGACTGATGCT; R:TGCCATTGCACAACTCTTTTC
Cd45 (SYBR Green primer)	Microsynth	F:ATGGTCCTCTGAATAAAGCCCA; R:TAGCACTATTGGTAGGCICC
ADIPOQ (SYBR Green primer for ChIP-qPCR)	Sigma-Aldrich	F:GGCTTTCACAATGTCACTGACTG;

		R:GCTGTAGCT ATTGCACAAGG TG
PPARG (SYBR Green primer for ChIP-qPCR)	Sigma-Aldrich	F:GATGTTTTGG GGCTTAATGGC A; R:TGGCTGGGT CTGAACATCAC

**Chemicals, peptides and recombinant proteins**		
Bovine Serum Albumin (fatty acid free)	Sigma-Aldrich	Cat#A6003
Bovine Serum Albumin	Sigma-Aldrich	Cat#A4503
Collagenase Type I (for WAT samples from *in vivo* experiments)	Worthington Biochemical Corporation	Cat#ILS004197
Collagenase from Clostridium histolyticum	Sigma-Aldrich	Cat#C0130
Insulin (*in vivo* treatments)	NovoNordisk	Actrapid HM
Recombinant Mouse IL-1β	R&D Systems	Cat#401-mL-010
^{3}H-2-deoxy-glucose	PerkinElmer	Cat#NET328A001 MC
Phosphatase inhibitor cocktail 2	Sigma-Aldrich	Cat#P5726
Poly-Prep® Columns, AG® 1-X8	Bio-Rad	Cat# 7316212
Ultima Gold	PerkinElmer	Cat#6013326
IL1Ra (*in vivo* treatments)	Sobi	Kineret®
Human recombinant IL-1β (*in vitro* treatments)	Sigma-Aldrich	Cat#I9401 or Cat#H6291
Human recombinant IFN-γ	Sigma-Aldrich	Cat#I17001
Human recombinant IL-6	Sigma-Aldrich	Cat#SCU0001
Human recombinant MCP-1	Sigma-Aldrich	Cat#SRP3109
Human recombinant TNF-α	Sigma-Aldrich	Cat#T6674
LPS	Sigma-Aldrich	Cat#L4524

Human recombinant IL-1Ra (*in vitro* treatments)	Sigma-Aldrich	Cat#SRP3084
U0126	Cell Signaling	Cat#9903
SB203580	Sigma-Aldrich	Cat#S8307
JNK Inhibitor VIII	EMD Millipore	Cat#420135
Hoechst 33342	Invitrogen	Cat#H3570
Bodipy™ 493/503	Invitrogen	Cat#D3922
HCS LipidTOX™ Red neutral lipid stain	Invitrogen	Cat#H34476
Pierce™ RIPA buffer	Thermo Scientific	Cat#89901
7-aminoactinomycin D	BD Biosciences	Cat#559925
Zombie Aqua™ Fixable Viability Kit	Biolegend	Cat#423102
DAPI Staining Solution	Miltenyi Biotec	Cat#130-111-579
D-[3-^{3}H]-glucose	PerkinElmer	Cat#NET331A001MC
OptiPhase HiSafe 3	PerkinElmer	Cat#1200.437
Chow Diet	Kliba Nafag	Cat#3436
High-Fat Diet	ssniff Spezialdiäten GmbH	Cat#E15742-34

**Critical Commercial Assays**		
RNeasy Lipid Tissue Mini Kit	Qiagen	Cat#74804
RNeasy Micro Kit	Qiagen	Cat#74004
RNeasy Plus Universal Mini Kit	Qiagen	Cat#73404
NucleoSpin RNA, Mini kit for RNA purification	Macherey-Nagel	Cat#740955
GoScript™ Reverse Transcriptase	Promega	Cat#A2801
iScript cDNA synthesis kit	Bio-Rad Laboratories	Cat#1708891
GoTaq® qPCR	Promega	Cat#A6001
iQ SYBR Green Supermix	Bio-Rad Laboratories	Cat#1708884
TaqMan Universal PCR Master Mix	Applied Biosystems	Cat#43-181-57

Click-iT™ Plus EdU Cell Proliferation Kit	Invitrogen	Cat#C10637
Pierce™ BCA Protein Assay Kit	Thermo Scientific	Cat#23227
MAGNA ChIP HiSens Chromatin Immunoprecipitation Kit	Merck	Cat#17-10460
eBioscience™ Foxp3/Transcription Factor Staining Buffer Set	Invitrogen	Cat#00-5523-00
Mix-n-Stain™ CF™ 647 Antibody Labeling Kit	Sigma-Aldrich	Cat#MX647S100
