# Supplementary material for: IL-1β promotes adipogenesis by directly targeting adipocyte precursors
Source: Nat Commun. 2024 Sep 11;15:7957. doi: 10.1038/s41467-024-51938-x (PMC11390900; doi:10.1038/s41467-024-51938-x)
Supplement: Supplementary file 3 — Reporting Summary [file 41467_2024_51938_MOESM3_ESM.pdf]

nature portfolio

Corresponding author(s): Daniel T. Meier
Jurga Laurencikiene

Last updated by author(s): May 2, 2024

Reporting Summary

Nature Portfolio wishes to improve the reproducibility of the work that we publish. This form provides structure for consistency and transparency in reporting. For further information on Nature Portfolio policies, see our Editorial Policies and the Editorial Policy Checklist.

Statistics

For all statistical analyses, confirm that the following items are present in the figure legend, table legend, main text, or Methods section.

n/a	Confirmed	
☐	☒	The exact sample size (*n*) for each experimental group/condition, given as a discrete number and unit of measurement
☐	☒	A statement on whether measurements were taken from distinct samples or whether the same sample was measured repeatedly
☐	☒	The statistical test(s) used AND whether they are one- or two-sided<br>*Only common tests should be described solely by name; describe more complex techniques in the Methods section.*
☐	☒	A description of all covariates tested
☐	☒	A description of any assumptions or corrections, such as tests of normality and adjustment for multiple comparisons
☐	☒	A full description of the statistical parameters including central tendency (e.g. means) or other basic estimates (e.g. regression coefficient) AND variation (e.g. standard deviation) or associated estimates of uncertainty (e.g. confidence intervals)
☐	☒	For null hypothesis testing, the test statistic (e.g. *F*, *t*, *r*) with confidence intervals, effect sizes, degrees of freedom and *P* value noted<br>*Give P values as exact values whenever suitable.*
☒	☐	For Bayesian analysis, information on the choice of priors and Markov chain Monte Carlo settings
☒	☐	For hierarchical and complex designs, identification of the appropriate level for tests and full reporting of outcomes
☐	☒	Estimates of effect sizes (e.g. Cohen's *d*, Pearson's *r*), indicating how they were calculated

Our web collection on statistics for biologists contains articles on many of the points above.

Software and code

Policy information about availability of computer code

Data collection	Flow cytometry - BD FACSDiva™ Software; Adiposoft (Fiji, ImageJ), HCS Studio: Cellomics Scan (v6.6.0) (Thermo Fisher Scientific).
Data analysis	IBM SPSS Statistics v28 (IBM SPSS, Armonk, NY); GraphPad Prism v9 (GraphPad Software, San Diego, CA); transcription factor motif activity changes was performed by ISMARA (PMID: 24515121); for RNA seq analysis, reads were assessed for quality by FastQC (v0.11.8) and aligned to the Ensembl GRCh38 reference genome using STAR (v2.6.1d). Gene counts were obtained using featureCounts (v1.5.1). Bioconductor package DESEq2 (v1.22.2) was used for normalization and sample group comparisons, generating log2 fold changes; for qPCR data analysis QuantStudio 5 (Applied Biosystems) was used. For Lipid droplet accumulation, image analysis software HCS Studio: Cellomics Scan (v6.6.0) (Thermo Fisher Scientific) was employed. For flow cytometry data analysis FlowJo v. 10.8 and FACS Diva software v. 7.0 were used; Adiposoft (Fiji, ImageJ);

For manuscripts utilizing custom algorithms or software that are central to the research but not yet described in published literature, software must be made available to editors and reviewers. We strongly encourage code deposition in a community repository (e.g. GitHub). See the Nature Portfolio guidelines for submitting code & software for further information.

Data

Policy information about availability of data

All manuscripts must include a data availability statement. This statement should provide the following information, where applicable:

- Accession codes, unique identifiers, or web links for publicly available datasets
- A description of any restrictions on data availability
- For clinical datasets or third party data, please ensure that the statement adheres to our policy

Data availability

RNA-seq data have been deposited at NCBI gene expression omnibus (GEO) under the accession number GSE237151. All data supporting the main findings are provided in the Source Data File and other data are available from the corresponding authors upon reasonable request. Additionally, previously published postprandial microarray data25 can be accessed at GEO under the accession number GSE142401. Gene expression data of scWAT stromal vascular cell populations and paired mature adipocytes81 is available at GEO under the accession number GSE100795. A CAGE dataset of hASCs at different time points of differentiation was previously collected86 within the FANTOM5 project. Clinical cohort data has been previously published43. Experimental data are included into a Source file and made available upon publication of the manuscript.

Research involving human participants, their data, or biological material

Policy information about studies with human participants or human data. See also policy information about sex, gender (identity/presentation), and sexual orientation and race, ethnicity and racism.

Reporting on sex and gender	In this study, only women cohorts were used. We state sex of individuals in the method section. Adipose-derived stem cells used in a study were obtained from a male donor, which is also stated in the Methods.
Reporting on race, ethnicity, or other socially relevant groupings	Individuals included into clinical cohort were mainly European origin (47 out of 56 had both parents born in European countries, one individual had one parent born in Middle East and information was lacking for the rest 8 individuals).
Population characteristics	The human cohort has been previously described and characteristics such as age, BMI, insulin sensitivity are provided wthithin that study. Reference 46 in the manuscript (Arner et al, Diabetes, 2012, PMID: 22688341)
Recruitment	Information on clinical cohort analyzed in this study is published (Arner et al, Diabetes, 2012, PMID: 22688341)
Ethics oversight	The information about ethical approval is provided in the manuscript.

Note that full information on the approval of the study protocol must also be provided in the manuscript.

Field-specific reporting

Please select the one below that is the best fit for your research. If you are not sure, read the appropriate sections before making your selection.

[x] Life sciences   [ ] Behavioural & social sciences   [ ] Ecological, evolutionary & environmental sciences

For a reference copy of the document with all sections, see nature.com/documents/nr-reporting-summary-flat.pdf

Life sciences study design

All studies must disclose on these points even when the disclosure is negative.

Sample size	Power calculation were used to guide animal numbers and a size of human cross-sectional cohort.
Data exclusions	No data was excluded.
Replication	As general rule, in vivo experiments were performed in 3 independent cohorts. All attempts at replication were successful. In vitro, experiments were performed at least in three biological replicates, each containing 2-3 technical replicates. n numbers are specified for each data panel
Randomization	In cohorts of mice, genotype and treatment were allocated randomly.
Blinding	In vivo studies involving injecting substances could not be done in a blinded manner. Mouse genotype was blinded during remaining in vivo studies. Data analysis was done in a blinded manner. In vitro experiments could not be performed in a blinded manner. The analysis was done in a blinded manner. Data collection was not human-dependent, performed by machines, therefore blinding was not relevant in most of the cases.

Reporting for specific materials, systems and methods

We require information from authors about some types of materials, experimental systems and methods used in many studies. Here, indicate whether each material, system or method listed is relevant to your study. If you are not sure if a list item applies to your research, read the appropriate section before selecting a response.

Materials & experimental systems

n/a	Involved in the study
☐	☒ Antibodies
☒	☐ Eukaryotic cell lines
☒	☐ Palaeontology and archaeology
☐	☒ Animals and other organisms
☐	☒ Clinical data
☒	☐ Dual use research of concern
☒	☐ Plants

Methods

n/a	Involved in the study
☒	☐ ChIP-seq
☐	☒ Flow cytometry
☒	☐ MRI-based neuroimaging

Antibodies

Antibodies used

All antibodies are listed in Supplementary table 1 and below. Vendors and catalog numbers are indicated.

Validation

Only commercially available antibodies were used in the study where validations were performed by the company. Control samples and fluorescence minus one (FMO) controls (in case of flow cytometry) were always included. Links to manufacturers' website with validations are provided below:

Anti-CD206-AF488 (Clone C068C2) (1:100) Biolegend Cat#141710 (https://www.biolegend.com/en-gb/products/alexa-fluor-488-anti-mouse-cd206-mmr-antibody-7426)
Anti-XCR1-PerCP-Cy5.5 (Clone ZET) (1:100) Biolegend Cat#148208 (https://www.biolegend.com/en-gb/products/percp-cyanine5-5-anti-mouse-rat-xcr1-antibody-10397)
Anti-CD317-BUV395 (Clone 927) (1:100) BD Biosciences Cat#747602 (https://www.bdbiosciences.com/en-be/products/reagents/flow-cytometry-reagents/research-reagents/single-color-antibodies-ruo/buv395-rat-anti-mouse-cd317-bst2.747602)
Anti-CD8-BUV496 (Clone 53-6.7) (1:100) BD Biosciences Cat#569181 (https://www.bdbiosciences.com/en-be/products/reagents/flow-cytometry-reagents/research-reagents/single-color-antibodies-ruo/buv496-rat-anti-mouse-cd8a.569181)
Anti-NK1.1-BUV563 (Clone PK136) (1:50) BD Biosciences Cat#741233 (https://www.bdbiosciences.com/en-be/products/reagents/flow-cytometry-reagents/research-reagents/single-color-antibodies-ruo/buv563-mouse-anti-mouse-nk-1-1.741233)
Anti-CD172a-BUV615 (Clone P84) (1:100) BD Biosciences Cat#751214 (https://www.bdbiosciences.com/en-be/products/reagents/flow-cytometry-reagents/research-reagents/single-color-antibodies-ruo/buv615-rat-anti-mouse-cd172a.751214)
Anti-CD49b-BUV661 (Clone HMa2) (1:200) BD Biosciences Cat#741523 (https://www.bdbiosciences.com/en-be/products/reagents/flow-cytometry-reagents/research-reagents/single-color-antibodies-ruo/buv661-hamster-anti-mouse-cd49b.741523)
Anti-CD45-BUV805 (Clone 30-F11) (1:100) BD Biosciences Cat#568336 (https://www.bdbiosciences.com/en-be/products/reagents/flow-cytometry-reagents/research-reagents/single-color-antibodies-ruo/buv805-rat-anti-mouse-cd45.568336)
Anti-F4/80-AF647 (Clone Cl:A3-1) (1:10) Bio-Rad Laboratories Cat#MCA497A647 (https://www.bio-rad-antibodies.com/monoclonal/mouse-f4-80-antibody-cl-a3-1-mca497.html?f=alexa%20fluor%C2%AE%20647)
Anti-MHCII-APC/Fire750 (Clone M5/114.15.2) (1:400) Biolegend Cat#107651 (https://www.biolegend.com/de-at/products/apc-fire-750-anti-mouse-i-a-i-e-antibody-13215)
Anti-CD64-BV421 (Clone X54-5/7.1) (1:50) Biolegend Cat#139309 (https://www.biolegend.com/de-at/products/brilliant-violet-421-anti-mouse-cd64-fcgammari-antibody-8992)
Anti-Ly6C-BV570 (Clone HK1.4) (1:100) Biolegend Cat#128030 (https://www.biolegend.com/de-at/products/brilliant-violet-570-anti-mouse-ly-6c-antibody-7392)
Anti-CD4-BV605 (Clone RM4-5) (1:50) Biolegend Cat#100547 (https://www.biolegend.com/de-at/products/brilliant-violet-605-anti-mouse-cd4-antibody-7627)
Anti-CD19-BV650 (Clone 6D5) (1:100) Biolegend Cat#115541 (https://www.biolegend.com/de-at/products/brilliant-violet-650-anti-mouse-cd19-antibody-7851)
Anti-CD11c-BV711 (Clone HL3) (1:25) BD Biosciences Cat#563048 (https://www.bdbiosciences.com/en-ca/products/reagents/flow-cytometry-reagents/research-reagents/single-color-antibodies-ruo/bv711-hamster-anti-mouse-cd11c.563048)
Anti-Ly6G-BV750 (Clone 1A8) (1:200) BD Biosciences Cat#747072 (https://www.bdbiosciences.com/en-ca/products/reagents/flow-cytometry-reagents/research-reagents/single-color-antibodies-ruo/bv750-rat-anti-mouse-ly-6g.747072)
Anti-CD49a-BV785 (Clone Ha31/8) (1:100) BD Biosciences Cat#740919 (https://www.bdbiosciences.com/en-ca/products/reagents/flow-cytometry-reagents/research-reagents/single-color-antibodies-ruo/bv786-hamster-anti-rat-mouse-cd49a.740919)
Anti-Siglec-F-PE (Clone E50-2440) (1:100) BD Biosciences Cat#562068 (https://www.bdbiosciences.com/en-ca/products/reagents/flow-cytometry-reagents/research-reagents/single-color-antibodies-ruo/pe-rat-anti-mouse-siglec-f.562068)
Anti-CD11b-PE/Dazzle594 (Clone M1/70) (1:200) Biolegend Cat#101256 (https://www.biolegend.com/fr-ch/products/pe-dazzle-594-anti-mouse-human-cd11b-antibody-9826)
Anti-CD3e-PE-Cy5 (Clone 145-2C11) (1:50) BD Biosciences Cat#553065 (https://www.bdbiosciences.com/en-ca/products/reagents/flow-cytometry-reagents/research-reagents/single-color-antibodies-ruo/pe-cy-5-hamster-anti-mouse-cd3e.553065)
Anti-TIM4-PE-Cy7 (Clone RMT4-54) (1:50) Biolegend Cat#130009 (https://www.biolegend.com/fr-ch/products/pe-cyanine7-anti-mouse-tim-4-antibody-11944)
Anti-CD16/CD32 (Clone 2.4G2) (Mouse BD Fc BlockTM) BD Biosciences Cat#553142 (https://www.bdbiosciences.com/en-ca/products/reagents/flow-cytometry-reagents/research-reagents/single-color-antibodies-ruo/purified-rat-anti-mouse-cd16-cd32-mouse-bd-fc-block.553142)

Anti-CD45-APC/Cy7 (Clone 30-F11) (1:1000) BioLegend Cat#103116 (https://www.biolegend.com/en-gb/products/apc-cyanine7-anti-mouse-cd45-antibody-2530)
Anti-CD31-APC/Fire 750 (Clone MEC13.3) (1:1000) BioLegend Cat#102528 (https://www.biolegend.com/en-gb/products/apc-fire-750-anti-mouse-cd31-antibody-15033)
Anti-CD142 (Polyclonal) (1:20) Novus Biologicals Cat#NBP2-15139 (https://www.novusbio.com/products/coagulation-factor-iii-tissue-factor-antibody_nbp2-15139)
Anti-CD54 (ICAM-1)-PE/Cy7 (Clone YN1/1.7.4) (1:100) BioLegend Cat#116122 (https://www.biolegend.com/en-gb/products/pe-cyanine7-anti-mouse-cd54-antibody-14759)
Anti-CD26 (DPP-4)-FITC (Clone H194-112) (1:200) BioLegend Cat#137806 (https://www.biolegend.com/en-gb/products/fitc-anti-mouse-cd26-dpp-4-antibody-6946)
FcR Blocking reagent, mouse (1:10) Miltenyi Biotec Cat#130-092-575 (https://www.miltenyibiotec.com/UN-en/products/fcr-blocking-reagent-mouse.html)

Anti-CD31-PE-Cy7 (Clone WM59) (1:12.5) BD Biosciences Cat#563651 (https://www.bdbiosciences.com/en-be/products/reagents/flow-cytometry-reagents/research-reagents/single-color-antibodies-ruo/pe-cy-7-mouse-anti-human-cd31.563651)
Anti-CD34-PE-CF594 (Clone 563) (1:250) BD Biosciences Cat#562449 (https://www.bdbiosciences.com/en-be/products/reagents/flow-cytometry-reagents/research-reagents/single-color-antibodies-ruo/pe-cf594-mouse-anti-human-cd34.562449)
Anti-CD45-AF700 (Clone HI30) (1:100) BD Biosciences Cat#560566 (https://www.bdbiosciences.com/en-be/products/reagents/flow-cytometry-reagents/research-reagents/single-color-antibodies-ruo/alexa-fluor-700-mouse-anti-human-cd45.560566)

CD45 MicroBeads, mouse (Clone 30F11.1) Miltenyi Biotec Cat#130-052-301 (https://www.miltenyibiotec.com/US-en/products/cd45-microbeads-mouse.html)
Anti-C/EBPd (Clone C-6) (Western Blot: 1:500; ChIP: 3 ug) Santa Cruz Biotechnology Cat#sc-365546 (https://www.scbt.com/p/c-ebp-delta-antibody-c-6)
Anti-C/EBPb (LAP) (Polyclonal) (Western Blot 1:1000) Cell Signaling Cat#3087 (https://www.cellsignal.com/products/primary-antibodies/c-ebpb-lap-antibody/3087)
Anti-Lamin A/C (Clone 4C11) (Western Blot 1:1000) Cell Signaling Cat#4777 (https://www.cellsignal.com/products/primary-antibodies/lamin-a-c-4c11-mouse-mab/4777)
Anti-P-CREB (S133) (Clone 87G3) (Western Blot 1:1000) Cell Signaling Cat#9198 (https://www.cellsignal.com/products/primary-antibodies/phospho-creb-ser133-87g3-rabbit-mab/9198)
Anti-CREB (Clone 48H2) (Western Blot 1:1000) Cell Signaling Cat#9197 (https://www.cellsignal.com/products/primary-antibodies/creb-48h2-rabbit-mab/9197)
Anti-rabbit IgG, HRP-linked (Western Blot 1:10000) Cell Signaling Cat#7074 (https://www.cellsignal.com/products/secondary-antibodies/anti-rabbit-igg-hrp-linked-antibody/7074)
Anti-mouse IgG, HRP-linked (Polyclonal) (Western Blot 1:10000) Invitrogen Cat#G21040 (https://www.thermofisher.com/antibody/product/Goat-anti-Mouse-IgG-H-L-Cross-Adsorbed-Secondary-Antibody-Polyclonal/G-21040)
Anti-C/EBPb (Polyclonal) (ChIP: 3 ug) Santa Cruz Biotechnology Cat#sc-150 (https://www.scbt.com/p/c-ebp-beta-antibody-c-19)
Normal Mouse IgG (Polyclonal) (ChIP: 3 ug) EMD Millipore Cat#12-371 (https://www.merckmillipore.com/SE/en/product/Normal-Mouse-IgG,MM_NF-12-371)
Normal Rabbit IgG (Polyclonal) (ChIP: 3 ug) EMD Millipore Cat#12-370 (https://www.merckmillipore.com/SE/en/product/Normal-Rabbit-IgG,MM_NF-12-370)

Animals and other research organisms

Policy information about studies involving animals; ARRIVE guidelines recommended for reporting animal research, and Sex and Gender in Research

Laboratory animals	Laboratory animals: Wild-type or genetically modified mice (mus musculus) were used. Adipocyte-specific deletion of IL1R1 (IL1R1AKO) was achieved by breeding homozygous IL1R1 floxed mice (IL1R1FF, JAX: 028398) with adiponectin-Cre transgenic mice (JAX: 010803). Littermate mice (IL1R1FF) were used as control. Male and female mice (8-12 week-old at the beginning of the experiment) were used, as described in the figure legend. Wild-type male (8 week-old at the beginning of the experiment) mice on a C57BL/6J genetic background (JAX: 000664) were used as control of IL1R1-deficient male (8 week-old at the beginning of the experiment) mice (IL1R1-KO, JAX: 003245). Prior to intercrossing, all animals were fully backcrossed (at least 10 generations) onto a C57BL/6J genetic background. Wild-type male mice on a C57BL/6N (8 week-old at the beginning of the experiment) genetic background were used during the experiments with IL-1Ra.
Wild animals	This study did not use wild animals
Reporting on sex	Most of the experiments wer performed with male mice. When female were used, they are reported separately and indicated in the figure legend.
Field-collected samples	This study does not contain filed-collected samples
Ethics oversight	All animal experiments were performed according to the Swiss veterinary law and were approved by Swiss authorities (cantonal veterinary office of Basel, approval 2511 and 2401).

Note that full information on the approval of the study protocol must also be provided in the manuscript.

Clinical data

Policy information about clinical studies

All manuscripts should comply with the ICMJEguidelines for publication of clinical research and a completedCONSORT checklist must be included with all submissions.

Clinical trial registration	No clinical trial was performed
Study protocol	Cross sectional study protocol was approved by Regional Ethical committee of Stockholm County council, 2009-1881, 2011-1002
Data collection	Data collection was performed according to approved protocol and described in a study Arner et al.2012 PMID: 22688341
Outcomes	Gene expression, fat cell size and morphology value n subcutaneous adipose tissue.

Plants

Seed stocks	n/a
Novel plant genotypes	n/a
Authentication	n/a

Flow Cytometry

Plots

Confirm that:

- [x] The axis labels state the marker and fluorochrome used (e.g. CD4-FITC).
- [x] The axis scales are clearly visible. Include numbers along axes only for bottom left plot of group (a 'group' is an analysis of identical markers).
- [x] All plots are contour plots with outliers or pseudocolor plots.
- [x] A numerical value for number of cells or percentage (with statistics) is provided.

Methodology

Sample preparation	Cryo-preserved human scWAT was thawn washed (0.5% BSA and 2 mmol/l EDTA in PBS), filtered through a 70 micrometer cell strainer and cell amount was calculated using Burker chamber.
Instrument	FACSAria™ Fusion instrument (BD Biosciences) equipped with 355, 405, 488, 561, and 633 nm lasers; FACSymphony A5 (equipped with five lasers); FACSCANTO 2 flow cytometer;
Software	FlowJo v. 10.8 and FACS Diva software v. 7.0
Cell population abundance	Cell population abundance for each cell population is indicated in a source file
Gating strategy	Gating strategy is provided in Supplemental materials and is based on Florescence Minus One (FMO) controls.

- [x] Tick this box to confirm that a figure exemplifying the gating strategy is provided in the Supplementary Information.
